# **Time-course of balance training-related changes on static and dynamic balance performance in healthy children**

**DOI:** 10.1186/s13104-024-06745-4

**Published:** 2024-03-19

**Authors:** Thomas Muehlbauer, Michael Giesen, Nele Roß, Simon Schedler, Mathew W. Hill

**Affiliations:** 1 Division of Movement and Training Sciences, Biomechanics of Sport, University of Duisburg- Essen, Gladbecker Str. 182, 45141 Essen, Germany; 2 Centre for Physical Activity, Sport and Exercise Sciences, Coventry University, Coventry, UK

**Keywords:** Postural control, Childhood, Intervention, Timing, Adaptations

## Abstract

**Objective:**

In healthy children, there is evidence of improvements in static and dynamic balance performance following balance training. However, the time-course of balance training-related changes is unknown. Thus, we determined the effects of balance training after one, three, and six weeks of exercise on measures of static and dynamic balance in healthy children (*N* = 44, 20 females, mean age: 9.6 ± 0.5 years, age range: 9–11 years).

**Results:**

Participants in the intervention group (2 × 25 min balance exercises per week) compared to those in the control group (2 × 25 min track and field exercises and soccer practice per week) significantly improved their static (i.e., by measuring stance time in the One-Legged Stance test) and dynamic (i.e., by counting step number in the 3-m Beam Walking Backward test) balance performance. Late effects (after 6 weeks) occurred most frequently followed by mid-term effects (after 3 weeks) and then early effects (after 1 week). These findings imply that balance training is effective to improve static and dynamic measures of balance in healthy children, whereby the effectiveness increases with increasing training period.

**Trial registration:**

Current Controlled Trials ISRCTN16518737 (retrospectively registered at 24th August, 2023).

## Introduction

There is evidence from original studies [1, 2] and a systematic review with meta-analysis [3] that balance training (BT) is effective to improve static and dynamic balance in healthy children. For instance, Walchli et al. [1] detected significantly decreased postural sway during the One-Legged Stance (OLS) test on a spinning top after five weeks of BT (2 times/week) in children aged 6–7 years and 11–12 years. Moreover, Schedler et al. [2] reported significantly increased reach distances for the Y Balance Test-Lower Quarter in 10–11 year-old children following eight weeks of BT (2 times/week). However, these studies used a pre-post-design separated by several weeks and do therefore not provide any insights into the time-course of balance training-related changes in youth. For an optimal planning of training schedules with limited time to promote postural control at school (during P.E.) or in sports clubs, however, it is important to know whether BT effects occur early on or only in the long-term.

In fact, the time-course of changes in balance with training has so far only been studied in older adults [4–6]. For instance, Alizadehsaravi et al. [4] compared balance robustness (duration of balancing) and balance performance (centre of mass velocity) after one and ten training sessions in adults aged ≥ 65 years. The authors already observed significant improvements in both measures after one session as well as after ten sessions. Yet, transferring these findings to children is not legitimate, as older adults are subject to age-related degeneration processes in the sensory and motor systems [7–9]. In addition, due to growth, maturation, and development, children do not yet have a fully developed postural control system [10, 11].

Therefore, the aim of the study was to investigate the time-course of balance training-related changes (i.e., after 1, 3, and 6 weeks of BT) on measures of static and dynamic balance in healthy children. We hypothesised that BT would result in balance improvements, which take place in as little as two sessions per week (i.e., after 1 week of BT).

## Methods

### Participants

An *a priori* power analysis (*f* = 0.25, α = 0.05, *1-β* = 0.80, *r* =.25, 2 groups, 2 assessments, drop-out rate of 20% due to reasons not attributable to treatment) revealed that a total sample size of *N* = 42 participants (i.e., *n* = 21 per group) would be sufficient to detect significant within-between interactions [12]. Therefore, 44 children were recruited and randomly assigned to the intervention (INT) group or control (CON) group (Table 1). All individuals participated voluntarily in the study and had no neurological or musculoskeletal impairment. None of the participants had prior experience with the applied balance tests. Written informed consent and subject’s assent were obtained from all participants before the start of the study. In addition, parent’s approval was obtained for minors.

**Table 1:** Means ± standard deviations for the demographic characteristics of the sample by group

Characteristic	INT-group (*n* = 22)	CON-group (*n* = 22)	*p*-value
Chronological age [years]	9.7 ± 0.5	9.6 ± 0.6	0.577
Age range [years]	9–10	9–11	–
Biological age [years from PHV]	-1.22 ± 2.32	-1.34 ± 2.24	0.864
Sex [f, m]	10/12	10/12	–
Body height [cm]	136.7 ± 6.7	135.9 ± 6.1	0.651
Body mass [kg]	33.6 ± 10.4	30.1 ± 4.7	0.161
Body mass index [kg/m²]	17.7 ± 3.8	16.3 ± 2.2	0.145

*CON* control; *INT* intervention; *f* female; *m* male; *PHV* peak height velocity

### Balance assessment

The same skilled assessors (graduated sport scientists) conducted the balance assessments before training (pre) and after one (post 1 = early effects), three (post 2 = mid-term effects), and six (post 3 = late effects) weeks of training. The timed OLS test was used to assess static balance. Participants were asked to stand on their non-dominant leg (determined by self-report) for as long as possible but for a maximum of 60 s with (a) eyes closed on firm ground (EC-FI = supressed vision/proprioception dominant), (b) eyes opened on foam (i.e., AIREX balance pad) ground (EO-FO = vision dominant/modified proprioception), and (c) eyes closed on foam ground (EC-FO = supressed vision and modified proprioception/vestibular dominant). Following a practice trial, one data-collection trial was executed, and the maximal stance time (s) during each condition was used for further analysis. The timed OLS test is valid (concurrent and discriminative) and reliable (moderate to excellent) in youth [13, 14].

The 3-m Beam Walking Backward test [15] was used to determine dynamic balance. Participants were asked to walk backward at a self-selected speed from the beginning to the end of wooden beams (length: 3 m; height: 5 cm) that differed in width (i.e., 6.0, 4.5, and 3.0 cm) for a minimum of eight steps. After one practice trial, two data-collection trials per beam width were performed. The number of steps for the data-collection trials was added up resulting in a maximum of 16 steps per beam width. The 3-m Beam Walk test is valid (content, construct, and criterion-related) and reliable (moderate) in youth [15].

### Interventions

Both groups trained for six weeks (i.e., 2 × 25 min per week on non-consecutive days) in a group scenario that was supervised by two graduated students. The participants of the INT-group performed static (e.g., bipedal/tandem/unipedal stance), dynamic (e.g., balancing for-/back-/sideward), proactive (e.g., weight shifting for-/back-/sideward and reaching for-/back-/sideward with one leg/arm), and reactive (e.g., push/pull while standing/walking) balance exercises during physical education. Two to three trials per exercise (each trial lasted 30–45 s) were conducted. The rest period between trials and exercises was 30 and 45 s, respectively. Training progression was achieved by a gradual increase of trial number and duration. The participants in the CON-group conducted three weeks of soccer practice followed by three weeks of track and field exercises.

### Statistical analyses

Data were analysed using Statistical Package for Social Sciences (version 27.0) and reported as group means ± standard deviations. After normal distribution was confirmed (i.e., Shapiro-Wilk tests), a 2 (group: INT-group, CON-group) × 4 (test: pre, post 1, post 2, post 3) repeated measures analysis of variance (ANOVA) was conducted to detect significant within-between interactions. GLM contrasts (type: simple) were analysed to investigate the time-course of changes from pre (means the reference category) to post 1, post 2, and post 3. Further, effect size (*η*_p_^2^) was calculated and reported as small (0.02 ≤ *η*_p_^2^ ≤ 0.12), medium (0.13 ≤ *η*_p_^2^ ≤ 0.25), or large (*η*_p_^2^ ≥ 0.26) [16]. The α-value was *a priori* set at *p* <.05.

## Results

### Static balance performance

**Fig. 1:**
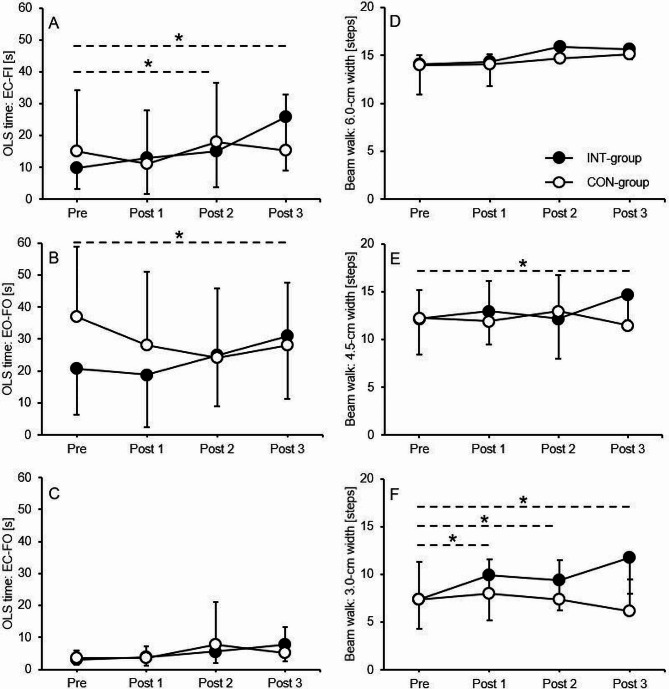
Early (post 1), mid-term (post 2), and late (post 3) effects of balance training on static balance performance using the One-Legged Stance test with (**A**) eyes closed on firm ground, (**B**) eyes opened on foam ground, and (**C**) eyes closed on foam ground and dynamic balance performance using the 3-m Beam Walking Backward test with (**D**) 6.0-cm beam width, (**E**) 4.5-cm beam width, and (**F**) 3.0-cm beam width. Values are means ± standard deviations. The asterisk (*) indicates a statistically significant difference (*p* <.05) compared to the pretest. *EC* eyes closed; *EO* eyes opened; *FI* firm ground; *FO* foam ground; *OLS* one-legged stance

For all but one (i.e., EO–FO = vision dominant/ modified proprioception) stance condition, the repeated measures ANOVA (Table 2) showed significant main effects of ‘Test’ (i.e., differences between the pretest and posttests, irrespective of group). ‘Group × test’ interactions (i.e., group-specific differences between the pretest and posttests) were detected for the EC–FI (supressed vision/proprioception dominant) and the EO–FO (vision dominant/ modified proprioception) stance conditions. GLM contrasts revealed significant mid-term and late improvements for the INT-group from pre to post 2 (EC–FI: *p* =.021, *η*_p_^2^ = 0.23) and post 3 (EC–FI: *p* <.001, *η*_p_^2^ = 0.55; EO–FO: *p* =.014, *η*_p_^2^ = 0.26) but not for the CON group (Fig. 1A–C). The main effect of ‘Group’ (i.e., differences between the INT-group and CON-group, irrespective of test) did not reach the level of significance, irrespective of stance condition.

**Table 2:** Early, mid-term, and late effects of balance training on static and dynamic balance performance in healthy children

Parameter	INT-group (*n* = 22)				CON-group (*n* = 22)				*p*-value (η_p_^2^)		
	Pre	Post 1 (early)	Post 2 (mid-term)	Post 3 (late)	Pre	Post 1 (early)	Post 2 (mid-term)	Post 3 (late)	Test	Group × Test	Group
*One-Legged Stance test*											
OLS time; EC–FI [s]	9.8 ± 6.5	13.0 ± 11.3	15.0 ± 11.3	25.7 ± 16.6	15.0 ± 19.2	11.2 ± 16.7	17.9 ± 18.7	15.4 ± 17.6	**< 0.001 (0.15)**	**0.002 (0.11)**	0.800 (0.01)
OLS time; EO–FO [s]	20.7 ± 14.3	18.8 ± 16.5	24.9 ± 15.9	30.9 ± 19.5	37.0 ± 21.8	28.1 ± 22.9	24.2 ± 21.7	28.0 ± 19.7	0.146 (0.04)	**0.009 (0.09)**	0.212 (0.04)
OLS time; EC–FO [s]	3.1 ± 1.7	3.7 ± 2.5	5.5 ± 3.6	7.8 ± 5.4	3.6 ± 2.2	3.6 ± 3.5	7.6 ± 13.5	5.2 ± 8.1	**0.008 (0.09)**	0.259 (0.03)	0.985 (0.01)
*3-m Beam Walk test*											
6.0-cm beam width [steps]	14.1 ± 3.2	14.3 ± 2.5	15.9 ± 0.4	15.6 ± 1.0	14.0 ± 2.8	14.1 ± 2.9	14.7 ± 2.3	15.1 ± 1.9	**0.007 (0.09)**	0.662 (0.01)	0.195 (0.04)
4.5-cm beam width [steps]	12.2 ± 3.8	13.0 ± 3.5	12.2 ± 4.2	14.7 ± 2.7	12.3 ± 2.9	11.9 ± 4.2	13.0 ± 3.7	11.5 ± 3.1	0.554 (0.02)	**0.007 (0.09)**	0.259 (0.03)
3.0-cm beam width [steps]	7.4 ± 3.1	9.9 ± 4.7	9.4 ± 3.2	11.7 ± 3.7	7.4 ± 3.9	8.0 ± 3.6	7.4 ± 4.1	6.1 ± 3.4	0.051 (0.06)	**< 0.001 (0.14)**	**0.006 (0.16)**

Data are presented as group mean values ± standard deviations. Values are *p*-values and effect sizes (*η*_p_^2^) in brackets with 0.02 ≤ *η*_p_^2^ ≤ 0.12 indicating small, 0.13 ≤ *η*_p_^2^ ≤ 0.25 indicating medium, and *η*_p_^2^ ≥ 0.26 indicating large effects. Bold values indicate statistically significant differences. The main effect of “Test” means differences between the pretest and posttests, irrespective of group. The main effect of “Group” means differences between the intervention and control group, irrespective of Test. The interaction effect of “Group × Test” means group-specific differences between the pretest and posttests. *EC* Eyes closed; *EO* Eyes opened; *FI* Firm ground; *FO* Foam ground; *OLS* One-Legged Stance test

### Dynamic balance performance

A significant main effect of ‘Test’ was only detected for the 6.0-cm beam width (Table 2). ‘Group × test’ interactions were observed for the 4.5-cm and 3-cm beam width. GLM contrasts revealed significant early, mid-term, and late improvements for the INT-group from pre to post 1 (3.0-cm: *p* =.013, *η*_p_^2^ = 0.26), post 2 (3.0-cm: *p* =.006, *η*_p_^2^ = 0.30), and post 3 (4.5-cm: *p* <.001, *η*_p_^2^ = 0.47; 3.0-cm: *p* <.001, *η*_p_^2^ = 0.52) but not for the CON group (Fig. 1D–F). A significant main effect of ‘Group’ was only found for the 3.0-cm beam width.

Please insert Fig. 1A–F about here.

## Discussion

In agreement with the first part of our hypothesis, we detected significant improvements in static (increased stance time in the OLS test) and dynamic (increased number of steps in the 3-m Beam Walking Backward test) balance for the INT-group compared to the CON-group. These findings are consistent with those from previous studies [1, 2] that investigated the effect of BT in healthy children and indicate that BT is an effective training regimen to enhance static and dynamic balance in this age group.

Late effects after six weeks of BT (i.e., 12 sessions) occurred most frequently followed by mid-term effects (after 3 weeks of BT = 6 sessions) and then early effects (after 1 week of BT = 2 sessions); confirming the second part of our hypothesis. This finding implies that BT is an effective approach to improve measures of static and dynamic measures of balance performance, whereby the effectiveness increases with increasing training period. Only for the most difficult stance condition (EC–FO) and the easiest walking condition (6.0-cm beam width), we did not detect significant improvements, indicating a ‘floor’ and ‘ceiling’ effect, respectively. Apparently, in the first case, the combination of standing with eyes closed on foam ground was too difficult, which limited the potential for enhancements. In the second case, the number of steps was already close to the maximum of 16, leaving little room for further improvements.

To our knowledge, this study is the first one that investigated the time-course of balance training-related changes in children. Consequently, the results can only be interpreted in the context of studies that examined different age groups. In previous publications on older adults, static and dynamic balance performance already improved after a single session of training [4, 5] but improved gradually over multiple sessions [4, 6]. In summary, these and our findings suggest that BT leads to improvements early on, but that the potential for adaptations can be further utilized with several training sessions. From a practitioner’s perspective, BT is an effective regimen for improving static and dynamic balance performance after only a few sessions and can therefore already be used with limited time resources. However, at least in children longer training periods seem to be beneficial to produce greater adaptations.

With regard to the underlying mechanisms, it can be speculated, based on studies with adults, that these take place at the spinal and supraspinal level [17]. Specifically, decreasing reflex activities [18, 19] as well as structural and functional brain changes [20, 21] were shown as a result of long- but also short-term periods of BT. Future studies should examine whether these mechanisms also occur in children and adolescents.

## Conclusion

This study investigated the early, mid, and late effects of BT in healthy children. We found significant improvements in static (i.e., increased stance duration in the OLS test) and dynamic (i.e., increased number of steps in the 3-m Beam Walking Backward) balance for the intervention compared to the active control group. Most frequently, late effects (after 6 weeks of training) occurred followed by mid-term (after 3 weeks of training) and then early effects (after 1 week of training). These results imply that BT is an effective training regimen in healthy children and the effectiveness of BT increases with increasing training period.

## Limitations

- Only children were investigated, which limits the generalisation of findings to adolescents.
- No laboratory-based testing (e.g., postural sway via force-plate) was applied, which limits the internal validity.
- The time-course of balance training-related changes was determined on a behavioural but not on a neuronal level (i.e., brain/muscle activation).

## Data Availability

The data generated and analysed during the present study are not publicly available due to ethical restrictions but are available from the corresponding author upon reasonable request.

## Declarations

### Author details

^1^Division of Movement and Training Sciences/Biomechanics of Sport, University of Duisburg-Essen, Essen, Germany.

^2^Centre for Physical Activity, Sport and Exercise Sciences, Coventry University, Coventry, United Kingdom.

## Abbreviations

ANOVA: Analysis of variance
BT: Balance training
CON: Control group
EC: Eyes closed
EO: Eyes opened
FI: Firm ground
FO: Foam ground
INT: Intervention group
OLS: One-legged stance test
PHV: peak height velocity
